# ‘All In’: a pragmatic framework for COVID‐19 testing and action on a global scale

**DOI:** 10.15252/emmm.202012634

**Published:** 2020-05-27

**Authors:** Syril D Pettit, Keith R Jerome, David Rouquié, Bernard Mari, Pascal Barbry, Yasunari Kanda, Mineo Matsumoto, Susan Hester, Leah Wehmas, Jason W Botten, Emily A Bruce

**Affiliations:** ^1^ Health and Environmental Sciences Institute Washington DC USA; ^2^ Virology Division Department of Laboratory Medicine University of Washington Seattle WA USA; ^3^ SAS Bayer Crop Science Sophia Antipolis France; ^4^ Institut de Pharmacologie Moléculaire et Cellulaire (IPMC) CNRS Université Côte d'Azur Valbonne France; ^5^ National Institute of Health Sciences Kanagawa Japan; ^6^ Pharmaceuticals and Medical Devices Agency Tokyo Japan; ^7^ Office of Research and Development Environmental Protection Agency Research Triangle Park NC USA; ^8^ Division of Immunobiology Department of Medicine Larner College of Medicine University of Vermont Burlington VT USA

**Keywords:** Microbiology, Virology & Host Pathogen Interaction, S&S: Ethics

## Abstract

Current demand for SARS‐CoV‐2 testing is straining material resource and labor capacity around the globe. As a result, the public health and clinical community are hindered in their ability to monitor and contain the spread of COVID‐19. Despite broad consensus that more testing is needed, pragmatic guidance toward realizing this objective has been limited. This paper addresses this limitation by proposing a novel and geographically agnostic framework (the 4Ps framework) to guide multidisciplinary, scalable, resource‐efficient, and achievable efforts toward enhanced testing capacity. The 4Ps (Prioritize, Propagate, Partition, and Provide) are described in terms of specific opportunities to enhance the volume, diversity, characterization, and implementation of SARS‐CoV‐2 testing to benefit public health. Coordinated deployment of the strategic and tactical recommendations described in this framework has the potential to rapidly expand available testing capacity, improve public health decision‐making in response to the COVID‐19 pandemic, and/or to be applied in future emergent disease outbreaks.

## Graphical abstract

This paper proposes a novel 4Ps Framework (Prioritize, Propagate, Partition, and Provide) to guide multidisciplinary, scalable, resource‐efficient, and achievable efforts towards enhanced SARS‐CoV‐2 testing capacity and improved public health decision‐making in response to the COVID‐19 pandemic.

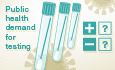

## Unmet needs

**Figure 1:**
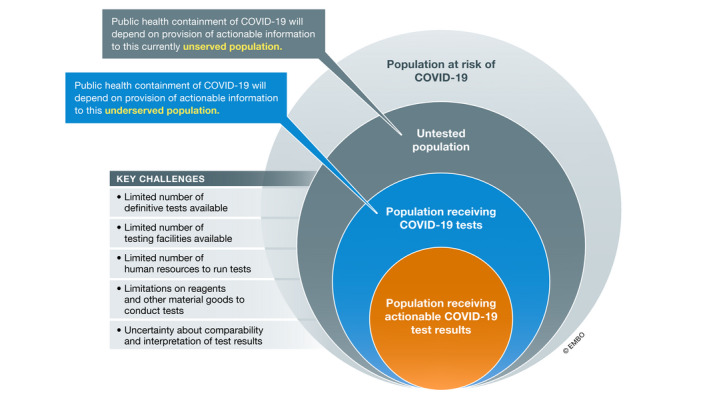
Critical gaps in population‐level COVID‐19 testing Illustration of contemporary challenges in providing testing to support public health containment of COVID‐19 and key populations that are currently unserved or underserved by testing.

If there is one element that the existing COVID‐19 pandemic response plans agree upon, it is the need for broadly accessible and implementable testing that enables case detection, contact tracing, and the “flattening of the curve” of the pandemic spread (McClellan *et al*, 2020; Organisation for Economic Co‐operation and Development, 2020). Testing, both for viral load (virologic testing) and for antibodies against SARS‐CoV‐2 developed by previously infected individuals (serologic assessment), will be fundamental for the “re‐opening” of public spaces and safe return of workforces in regions where SARS‐CoV‐2 is already widespread and to track and contain emergence/re‐emergence where the prevalence of the virus is lower. From February 2020 through the date of this publication, the global public and private sectors have invested in the development and distribution of new affordable and accessible tests, sample collection swabs, and reagents for clinical testing centers. Regulatory agencies such as the US FDA provided added regulatory flexibility to promote rapid adoption of new methods. Despite these efforts, the capacity for testing and test processing (at local, national, and global levels) is failing to meet current and anticipated needs. The reasons are diverse and include lack of availability of test kits or components, limited labor to run the tests, limited number of testing facilities, shortages or uneven distribution of consumables and reagents for processing, shortages in swabs and personal protective equipment for sampling, and lack of clarity about how to interpret or act on a test result. Many of these limitations stem from a lack of local and/or global cooperation. The net impact of these limitations is that a substantial portion of the population that could/should be tested will not be tested. Additionally, and perhaps just as concerning, limited virologic test availability has delayed timelines for testing. As viral shedding appears to happen most significantly in the earlier stages of infection, unduly delayed testing can result in a higher false‐negative rate depending on a given test's sensitivity. The unfortunate synergy of these factors impacts both the ability for a clinician to advise an individual patient and for governments and health agencies to monitor and manage their population health. The ability to make informed decisions about personal or public health protection has been untenably affected by a lack of data on personal/local risk as well as regional caseloads and transmission rates (Fig 1). Concurrently, untapped capacities for testing or analysis in public and private sectors at a global scale are available and justify the pragmatic proposals discussed in this paper.

Just as the reasons for sampling and testing insufficiency are multifaceted and expansive, so too must be our solutions for addressing them. To further stem the spread of COVID‐19, a series of systematic and innovative implementation approaches to the design and execution of *virologic* testing are necessary. Such approaches should be tailored for broad adoption (or ready adaptation) as a means to simultaneously, comprehensively, and pragmatically address current hurdles to providing public access to adequate testing for COVID‐19. As a first step, the proposals outlined here focus primarily on the role of *virologic* testing as a coordinated first line of assessment with the recognition that serologic testing will ultimately play a critical role in longer‐term monitoring and control measures.

This communication defines four key pillars for action and a supporting conceptual model of implementation to promote a scalable and “all in” approach to virologic testing. We offer these pillars as an inclusive approach, a set of “this AND that” (not “this OR that”) considerations for enriching the breadth and actionability of COVID‐19 testing. Global progress against COVID‐19 will require that the pillars for action described below are pursued in parallel by relevant government and public health bodies whenever possible—not as alternatives.

The authors recognize that many state, regional, and national entities are actively developing approaches to bring more testing to their populations. The uniqueness of our approach lies in four simultaneous elements. Specifically, this approach:

• Is adaptable for use at a local or international level;
• Is not specific to any one health care or public health system or structure;
• Addresses testing accuracy and processing capacity as well as test availability; and
• Proposes efficient and pragmatic approaches to testing implementation and subsequent public health action.

This proposal reflects the input of a multi‐sector team of scientists with experience in regulatory, public health, clinical medicine, virology, molecular biology, and diagnostics arenas with recognition that successful implementation will require coordination and collaboration across broad spectrums of the scientific, medical, epidemiologic, and public health communities.

## Pillars for action

The following four pillars for action (*the 4Ps*) are offered to guide both comprehensive action and concrete action to enhance test availability and application (Box 1). As noted above, changes in the availability of actionable testing results will require effort toward *each of these areas in parallel* and with broad global cooperation.

**Figure 2:**
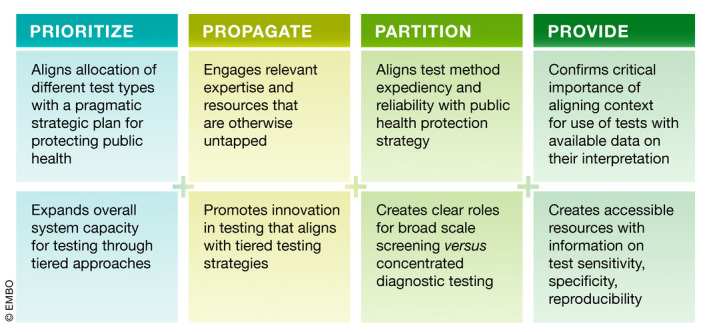
Elements of the 4Ps Framework Summary of key contributions of each of the four pillars of action.

A brief description of the rationale and proposed effort linked to each of these pillars follows below and is summarized in Fig 2. Selected references are included within the text, but additional resources in relation to these pillars and their implementation are available in Box 2.

#### Prioritize

The Prioritize pillar seeks to reconcile broad public health needs with pragmatic limitations in test availability. Quite simply, there are not enough diagnostic tests, diagnostic testing workforce, or diagnostic test cost coverage to test the millions of individuals potentially exposed to SARS‐CoV‐2 on a daily basis. This reality has led multiple groups—including ours—to propose and enact practices to optimize COVID‐19 diagnostic test availability for prioritized critical subpopulations. To achieve this objective, relevant public health officials must clearly define high priority populations and ensure that that the tests provided to those populations are adequately sensitive (using information developed via the *“*Provide” section below). Prioritization should involve temporally/situationally fluid conversations as to how broadly or restrictively to define a “critical” population eligible for diagnostic testing (e.g., front‐line health care workers, symptomatic patients, police/fire, co‐morbid populations, hospital in‐patients, essential workers, and children/teachers returning to schools). For example, over‐restriction of accreditation/permission for sampling and priority test eligibility has led to an underutilization of available diagnostics in some regions (e.g., in parts of the United States and France) in recent weeks.

While the pragmatic rationale for prioritization is clear, this approach leaves a significant portion of the population totally untested. It means that our understanding of population disease dynamics must rely almost exclusively on visible symptom reports rather than molecular screening, despite increasing evidence of asymptomatic and pre‐symptomatic transmission of COVID‐19. Therefore, we propose to enrich this prioritization strategy with *concurrent high*‐*throughput screening* for those segments of the population that do not qualify for diagnostic prioritization. The use of broad scale, non‐diagnostic, screening could provide an added data source (albeit imperfect) to inform the selection of future “priority” populations for diagnostic screening. With appropriate consent mechanisms, this screening could be used by health officials to flag individuals for subsequent diagnostic testing. While it may still be infeasible to capture 100% of a population (even with segmentation into diagnostic and screening testing)—it is anticipated that this approach would add to overall knowledge by screening individuals that might otherwise have zero actionable information around their health status (see the “Partition” section below). The “Prioritize” approach described here will be critical to expand our ability to understand and address COVID‐19's spread.

#### Propagate

The Propagate pillar has two core components. The first is to employ an “all hands” approach to advance the use of test methods that are both amenable for screening purposes *and* can be implemented using resources and expertise that might otherwise go untapped in this crisis. For example, a growing number of independent academic laboratories have begun developing flexible approaches to address different “chokepoints” in providing diagnostic testing. The current RT*–*PCR test that comprises the backbone of testing efforts requires RNA to be extracted from a patient sample, which is then reverse transcribed (RT) and amplified by polymerase chain reaction (PCR) to detect small amounts of viral genetic material with great specificity and sensitivity. The kits required to perform the RNA extraction step have been in particularly short supply worldwide; however, recent work that eliminates the RNA extraction step altogether and yet seems to retain sufficient sensitivity for screening purposes provides a potential way out of this reagent shortage (preprint: Beltran‐Pavez *et al*, 2020; preprint: Bruce *et al*, 2020; preprint: Smyrlaki *et al*, 2020). If sufficiently characterized for *screening,* this and other newly developing approaches could allow for more rapid and less reagent intensive virologic analysis of swabbed samples than the standard RT–PCR testing approaches that have been broadly employed for COVID testing (e.g., the majority of in vitro diagnostic tests receiving an Emergency Use Authorization (EUA) approval from the US FDA and/or are recommended by health authorities in other countries rely on RT–PCR methods with an RNA extraction step). Some of these labs are also exploring means to partially or totally inactivate the virus just after swab collection in order to increase the throughput of these more rapid RT*–*PCR methods and expand the number of labs eligible to process samples (preprint: Lista *et al*, 2020). Optimization of sampling procedures via protocols for alternative swabs and sampling media may also contribute to the propagation of testing via rapid RT*–*PCR or other means. For example, recent work indicates that saliva samples may be as sensitive as the nasopharyngeal swabs currently in use, while having the advantage of being much easier to collect and requiring less personal protective equipment and fewer key consumables such as swabs (Williams *et al*, 2020).

Other emerging strategies to expedite processing include pooling samples to allow for wider coverage of the population for screening or the use of rapid and inexpensive loop‐mediated isothermal amplification (LAMP) methods (preprint: Ben‐Ami *et al*, 2020; preprint: Schmid‐Burgk *et al*, 2020). In fact, the use of pooled samples (in which multiple patient samples are analyzed concurrently) is underway in some German health laboratories. For pools with a positive sample, subsequent diagnostic testing conducted on parallel reserved samples can be completed in rapid succession, whereas negative pools are not assessed further. Such approaches have the potential to enhance processing capacity by an order of magnitude—potentially without significant losses of sensitivity. Collectively, these alternative approaches could not only alleviate testing burden on diagnostic laboratories but also foster alternative methods that work in concert to help mitigate diversion of critical test reagents from diagnostic facilities.

This pillar's focus on expanding screening rather than diagnostic testing is intentional. Screening is considered “research” in most settings and thus not subject to the same regulatory and licensing standards as are mandated for diagnostic testing facilities. Evolving rapid and low‐resource RT*–*PCR SARS‐CoV‐2 testing approaches are offered here as examples of an opportunity to efficiently expand screening capacity by leveraging hardware and technicians that are (i) already in place and experienced in a broad range of different academic, government, and private sector laboratories around the globe and (ii) might otherwise be unable to support COVID‐19 testing efforts. Implementation of any novel screening tests would require that participating laboratories have appropriate biological safety experience/licensing for handling of viral samples. Even if used “only for screening” further scientific consensus on the execution, reliability, sensitivity, and interpretation of novel methods (like rapid RT‐PCR) will be required (i.e., “Provide” pillar). Collaboration with clinical facilities that have a range of relevant patient samples will be important for optimizing protocols to accommodate real‐world variability. Opportunities to rapidly build this consensus through collaborative networks of research scientists, clinicians, public health officials, and/or regulators should be pursued. Protocols for sample collection/patient consent and sample tracking and dissemination of test results to clinicians or public health officials in a timely manner while maintaining adequate privacy protections would also need to be developed and carefully monitored.

A second potential avenue for realizing this pillar is to expand *diagnostic* testing capacity by expanding the throughput of existing clinical diagnostic laboratories and/or newly engaging laboratories not typically involved in clinical testing. On the expansion side, some large and well‐funded academic centers (e.g., The Broad Institute, University of California at Berkeley) have been able to rapidly scale their research operations and obtain clinical diagnostic certification (CLIA) by partnering with on‐campus health partners to adapt to high‐throughput diagnostic testing demands during the pandemic. Financial and staffing constraints may limit the broad replicability of this approach, but when feasible it can be highly impactful.

We also propose active exploration of opportunities to expand the number of clinical diagnostic laboratories via novel interim or permanent designations to permit testing (e.g., special waivers and novel oversight programs). Some countries—such as Germany and the United Kingdom—have recently adopted novel processes for acceptance of diagnostic tests from non‐diagnostic laboratories to aid in the COVID testing response. Germany has also reported successes by decentralizing testing and thus leveraging a diversity of academic, industrial, and clinical sites throughout the country for testing, and laboratories can currently be accredited as diagnostic labs after reporting required results following a validation plan and a visit by a responsible (local) authority. These approaches could also be replicated in other countries to expand diagnostic capacity. For example, a team of clinical diagnostic testing experts (*e*.g., from the College of American Pathologists (CAP) in the U.S. or other regionally relevant certifying groups) could be engaged to perform a “peer review” of newly created non‐clinical COVID‐19 testing sites. These inspections could provide some level of confidence that the new sites can perform tests and produce valid results. Such inspections might be followed up with assessment of performance on positive/negative control samples to facilitate cross‐lab comparisons. The rapid incorporation of novel diagnostic labs (that lack prior experience in this space) would also require careful attention to record keeping around custody of samples and communication of results to clinicians and patients. Collaboration with existing testing facilities may facilitate implementation of pragmatic approaches in this regard.

In sum, the Propagate pillar offers concrete opportunities to expand capacity for screening and diagnostic testing. While such approaches may not be easily scalable or adaptable to all regulatory or economic settings, current successes in this arena evidence potential opportunity that should be actively explored.

#### Partition

This pillar calls for the contemporary integration of strategic and technical factors to partition tests into categories to communicate whether they are most appropriate for a diagnostic or screening context of use in a given setting. As described above (*Prioritize*), exhaustive diagnostic testing is not currently pragmatic whereas appropriately designed and characterized screening tests could and should be implemented *at scale* to provide more insights into population‐level spread. The designation of diagnostic vs. screening must be driven by available information on the biological sensitivity/specificity and technical reproducibility of a given test (generated via the “Provide” pillar) in combination with other situational considerations. For example, local testing or processing cost, accessibility, and processing time may factor into whether a test may be suitable for diagnostic or screening purposes. Previously published frameworks that guide considerations for assessing a diagnostic or screening test's fitness for purpose may be useful guides (Kosack *et al*, 2017). In designating a test as diagnostic vs. screening, it will also be essential to clearly communicate the actionable health measures that are to be aligned with either a positive or negative test outcome. These designations are anticipated to be fluid and may change over time or according to geographic variants in test availability, public health need, test precision, cost, and testing capacity.

#### Provide

The Provide pillar is rooted in the need for accessible and comparable information about test quality, accuracy, and specificity of tests used to support the Partition and Prioritize pillars above. While the availability of comparative characterizations of diagnostic (or screening) tests can be a challenge in clinical medicine and public health in general, it is essential that some evidence toward comparability be developed for purposes of efficiently implementing the 4Ps strategy. For example, consensus on a minimum set of characterizing criteria (e.g., limit of detection, reproducibility, comparing results with a publicly accessible set of standards, and sensitivity in comparison with the “standard” method including prior RNA isolation) could be defined. This type of approach has been previously employed to positive end to characterize nonclinical testing methods (e.g., for human lymphocyte activation assays) (Collinge *et al*, 2020). Testing kit manufacturers or test and instrument providers could be asked to voluntarily provide data against these minimal criteria in a publicly accessible online resource. As in all elements of this strategy, the perfect cannot be the enemy of the good. The development of comprehensive, independently evaluated, comparative datasets is neither feasible nor needed at this time. However, meaningful implementation of a testing and containment strategy across the globe will require due attention to the assessment of test quality and interpretation. Failure to focus on comparability of test results and methods will inhibit informed epidemiologic modeling of the spread of COVID‐19 as well as consistent and data‐driven clinical interventions.

## Implementing the 4Ps

The Pillars described above are offered as coordinated and cohesive strategic and tactical opportunities to meet many of the major challenges to broadened test implementation for SARS‐CoV‐2. As detailed above, the Pillars are proposed as interdependent and parallel work streams that require cross‐fertilization of data, effort, or strategy to be effective. Specific implementation of these approaches will require consideration of the social, economic, and especially legal or regulatory constraints relevant to the setting in which the framework is to be applied (i.e., local, regional, national, international). Detailed implementation scenarios are intentionally omitted here to allow for adaptation of this framework in a range of settings.

**Figure 3:**
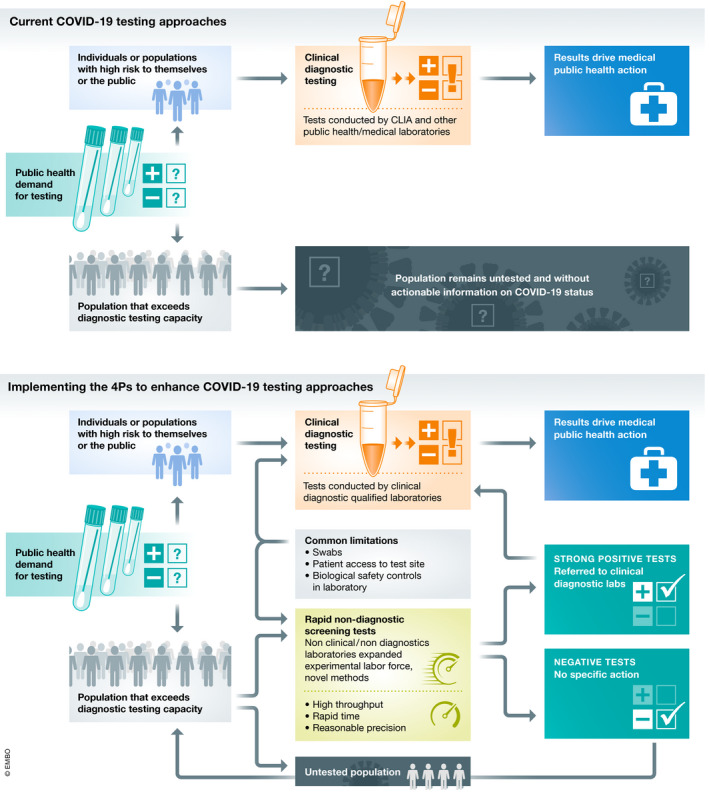
Applying 4Ps to bridge the gap Conceptual model of the application of the 4Ps framework to bridge the gap on COVID‐19 testing.

A broad conceptual model for the integration of the 4Ps to realize systemic change in test prioritization and actionability is illustrated in Fig 3. The tests, data, facilities, labor, and insights generated via the 4Ps can support enhanced diagnostic testing of critical populations and iteratively engage at least a portion of the otherwise untested community via screening. Implementing this model will require robust cooperation across stakeholders including public health authorities (to provide systemic oversight and to manage appropriate actions in response to screening/diagnostic evidence), the medical community (providing treatment for and management of confirmed positive patients), test centers and clinics (collecting and disseminating biological samples and ensuring appropriate patient consent and privacy), and research scientists (refining testing approaches and generating data on biological and statistical interpretation of screening and diagnostic tests). The proposed approaches offer an iterative, achievable, and flexible opportunity to meaningfully expand the percentage of the public engaging in some type of testing (diagnostic or screening) relative to the status quo. Although this approach would not capture 100% of a population via diagnostic or screening tests, the added system capacity could still have significant impacts on public health management of COVID‐19. The model aligns with and complements other emerging proposals around “local random testing” to address comprehensive testing limits (Kaplow, 2020). Depending upon the local situation and resources, the percent of a population to be engaged via diagnosis, screening, or neither could be actively modulated by health authorities. Decisions on feasible and legally/ethically permissible action following a confirmed positive diagnostic test (e.g., quarantine and contact tracing) will also impact COVID‐19 containment. While these are critical elements, they may be highly variable around the globe and are beyond the scope of this paper.

## Limitations

The 4Ps framework—like all efforts to address the complex challenges posed by COVID‐19—has many limitations and challenges. While this framework aims to be pragmatic and achievable, its potential for success will depend upon a rapid investment of time and commitment to a flexible, coordinated, and reinforced strategy by a range of small and large public health entities with the resources and influence to impact broad populations. Whether such commitments can be garnered in due time (or at all) is uncertain. Additionally, the strategy is offered as an “*and not or”* approach. The authors believe that such ambition is requisite for (and achievable in) the current global situation. However, its breadth of scope can be intimidating. Further systemic challenges to the successful roll out of this approach may come from existing (pre‐COVID) barriers to equitable access to care linked to social determinants of health. Early data suggest that morbidity and mortality from COVID‐19 are higher in minority communities with low socio‐economic status. Thus, the locational, cultural, temporal, and economic accessibility of testing will be a critical consideration for implementation. At a more granular level, challenges could arise in incentivizing or resourcing the development and dissemination of data that characterize novel tests or newly initiated labs, identifying test centers with adequate biological safety or clinical testing certifications, building data exchange (to support both clinical action and broad interventional strategies), or building or “enforcing” consistency of approach in responding to a diagnostic or screening test result. Despite these hurdles, the authors believe that progress toward the 4Ps is both essential and achievable.

## Path forward

The current lack of a coordinated and scalable approach to testing for SARS‐CoV‐2 poses an unsustainable hurdle to containment of COVID‐19 and blocks the reintroduction/sustenance of critical social, health, and economic functions worldwide. The 4Ps framework is offered as one example of a flexible but cohesive guide for tackling the global need for nimble test development and implementation strategies in response to COVID‐19 (or potentially future emergent viruses). This framework uniquely identifies opportunities to leverage expertise and resources already trained, available, and in many cases eager to contribute. Whether or not this specific framework is adopted, we call upon those with expertise, commitment, and remit to do all they can to endorse, realize, and build upon these essential elements for progress and help restore global public health.

## Conflict of interest

The views expressed in this paper are those of the authors and do not necessarily reflect the views or policies of the U.S. Environmental Protection Agency, Japan National Institute of Health Sciences, the Pharmaceuticals and Medical Devices Agency, or that of any of the authors’ employers. The authors have no conflicts to disclose.

**Box 1: The 4Ps toward test availability and actionability**

*Prioritize* diagnostic testing for individuals and populations most at risk of infection or at risk of infecting others.

*Propagate* testing capacity by expanding available test and sampling methods, as well as potentially expanding options for testing at non‐traditional laboratory venues.

*Partition* tests into screening vs. diagnostic applications to clearly delineate appropriate contexts of use.

*Provide* evidence‐based standards for characterizing test sensitivity, precision, and utility and apply them to available tests.

**Box 2: Further reading list**

**Evolving testing and sampling approaches for SARS‐CoV‐2**

Alcoba‐Florez J, Gonzalez‐Montelongo R, Inigo‐Campos A, Garcia‐Martinez de Artola D, Gil‐Campesino H, Ciuffreda L, *et al* Fast SARS‐CoV‐2 detection by   RT‐qPCR in preheated nasopharyngeal swab samples. *medRxiv*. 2020:2020.04.08.20058495. https://doi.org/10.1101/2020.04.08.20058495

Brown JR, Atkinson L, Shah D, Harris K, Prevention I, Kingdom U (2020). Validation of an extraction‐free RT‐PCR protocol for detection of SARS‐CoV2 RNA.   *medRxiv*. 2020.04.29.20085910. https://doi.org/10.1101/2020.04.29.20085910

Druce J, Garcia K, Tran T, Papadakis G, & Birch C (2012). Evaluation of swabs, transport media, and specimen transport conditions for optimal detection   of viruses by PCR. *Journal of Clinical Microbiology*. https://doi.org/10.1128/jcm.06551-11

Fomsgaard AS, & Rosenstierne MW (2020). An alternative workflow for molecular detection of SARS‐CoV‐2‐escape from the NA extraction kit‐shortage.   *medRxiv*. https://doi.org/10.1101/2020.03.27.20044495

Grant PR, Turner MA, Shin GY, Nastouli E, & Levett LJ (2020). Extraction‐free COVID‐19 (SARS‐CoV‐2) diagnosis by RT‐PCR to increase capacity for national   testing programmes during a pandemic. *bioRxiv*, 19, 2020.04.06.028316. https://doi.org/10.1101/2020.04.06.028316

He X, Lau EH, Wu P, Deng X, Wang J, Hao X, Lau YC, Wong JY, Guan Y, Tan X, *et al* (2020). Temporal dynamics in viral shedding and transmissibility of   COVID‐19. *Nature Medicine*. https://doi.org/10.1101/2020.03.15.20036707

Jazin EE, Cavelier L, Eriksson I, Oreland L, Gyllensten U, & Loeb LA (1996). Human brain contains high levels of heteroplasmy in the noncoding regions of   mitochondrial DNA. *Proceedings of the National Academy of Sciences*, 93(22), 12382–12387. https://doi.org/10.1073/pnas.93.22.12382

Lista MJ, Page R, Sertkaya H, Matos PM, Ortiz‐Zapater E, Maguire TJA, Poulton K, O'byrne AM, Bouton C, Dickenson RE, *et al* (2020). Resilient SARS‐CoV‐2   diagnostics workflows including viral heat inactivation. *medRxiv*. https://doi.org/10.1101/2020.04.22.20074351

Lohse S, Pfuhl T, Berkó‐Göttel B, Rissland J, Geißler T, Gärtner B, … Smola S. (2020). Pooling of samples for testing for SARS‐CoV‐2 in asymptomatic   people. *The Lancet Infectious Diseases*, 0(0). https://doi.org/10.1016/s1473-3099(20)30362-5

Rabe BA, & Cepko C (2020). SARS‐CoV‐2 Detection Using an Isothermal Amplification Reaction and a Rapid, Inexpensive Protocol for Sample Inactivation   and Purification. *medRxiv*. https://doi.org/10.1101/2020.04.23.20076877

Sentmanat M, Kouranova E, Cui X. One‐step RNA extraction for RT‐qPCR detection of 2019‐nCoV. *bioRxiv*. 2020:2020.04.02.022384. https://doi.org/10.1101/2020.04.02.022384

Shenta N, Levy S, Wuvshet V, & Skorniakov S (2020). Efficient high throughput SARS‐CoV‐2 testing to detect asymptomatic carriers. *medRxiv*. https://doi.org/10.1101/2020.04.06.025635

Smyrlaki I, Ekman M, Papanicolaou N, & Lentini A (2020). Massive and rapid COVID‐19 testing is feasible by extraction. *medRxiv*. https://doi.org/10.1101/2020.04.17.20067348

Srivastan S, Han P, van Raay K, Wolf C, McCulloch D, & Al E. (2020). Preliminary support for a “dry swab, extraction free” protocol for SARS‐CoV‐2 testing   via RT‐qPCR. *bioRxiv*, 1–11. https://doi.org/10.1101/2020.04.22.056283

Wölfel R, Corman VM, Guggemos W, Seilmaier M, Zange S, Müller MA, Niemeyer D, Jones TC, Vollmar P, Rothe C, *et al* (2020). Virological assessment of   hospitalized patients with COVID‐2019. *Nature*. https://doi.org/10.1038/s41586-020-2196-x

Wyllie AL, Fournier J, Casanovas‐Massana A, Campbell M, Tokuyama M, Vijayakumar P, Geng B, Muenker MC, Moore AJ, Vogels CBF, *et al* (2020). Saliva is   more sensitive for SARS‐CoV‐2 detection in COVID‐19 patients than nasopharyngeal swabs. *medRxiv*. https://doi.org/10.1101/2020.04.16.20067835

Yu L, Wu S, Hao X, Li X, Liu X, Ye S, Han H, Dong X, Li X, Li J, *et al* (2020). Rapid colorimetric detection of COVID‐19 coronavirus using a reverse tran‐  scriptional loop‐mediated isothermal amplification (RT‐LAMP) diagnostic platform: iLACO. *medRxiv*. https://doi.org/10.1101/2020.02.20.20025874

Zhang Y, Odiwuor N, Xiong J, Sun L, Nyaruaba RO, Wei H, & Tanner NA (2020). Rapid molecular detection of SARS‐CoV‐2 (COVID‐19) virus RNA using   colorimetric LAMP. *medRxiv*, 2, 2020.02.26.20028373. https://doi.org/10.1101/2020.02.26.20028373

**Transforming research sites for testing**

Bayer. (2020). Bayer to boost Germany's COVID‐19 analysis capacity by several thousand tests per day. https://media.bayer.com/baynews/baynews.nsf/id/Bayer-to-boost-Germanys-COVID-19-analysis-capacity-by-several-thousand-tests-per-day

Broad Institute. (2020). How Broad Institute converted a clinical processing lab into a large‐scale COVID‐19 testing facility in a matter of days. https://www.broadinstitute.org/news/how-broad-institute-converted-clinical-processing-lab-large-scale-covid-19-testing-facility

Hockemeyer D, Urnov F, Doudna JA, Amen AM, Barry K, Boyle JM, Brook CE, Choo S, Cornmesser LT, Dilworth DJ, *et al* (2020). Blueprint for a pop‐up   SARS‐CoV‐2 testing lab. *medRxivV*. https://doi.org/10.1101/2020.04.11.20061424

Sridhar S, Forrest S, Kean I, Young J, Scott JB, Maes M, … Baker S (2020). A blueprint for the implementation of a validated approach for the detection of   SARS‐Cov2 in clinical samples in academic facilities. *bioRxiv*. 2020.04.14.041319. https://doi.org/10.1101/2020.04.14.041319
